# Patient and provider experiences with PEN-Plus in rural Mozambique: cohort characteristics at baseline

**DOI:** 10.1080/16549716.2026.2623347

**Published:** 2026-03-02

**Authors:** Edi Fulai, Chantelle Boudreaux, Laura Drown, Whitney Puetz, Maryam Mansoor, Unícia Nyanula, Valeria Chicamba, Yolanda Marcelino, Emílio Tostão, Gene Bukhman, Ana O. Mocumbi, Alma J. Adler

**Affiliations:** a Departmento de Pesquisa Formação e Inquéritos de Saúde, Instituto Nacional de Saúde, Sofala, Mozambique; b Center for Integration Science in Global Health Equity, Brigham and Women’s Hospital, Boston, MA, USA; c Universidade Eduardo Mondlane, Maputo, Mozambique; d Department of Economics and Agricultural Development, Universidade Eduardo Mondlane, Maputo, Mozambique; e Department of Global Health and Social Medicine, Program in Global NCDs and Social Change, Harvard Medical School, Boston, MA, USA; f Instituto Nacional de Saúde, Maputo, Mozambique

**Keywords:** Noncommunicable diseases, healthcare service delivery, health equity, care model, global health

## Abstract

**Background:**

Severe chronic noncommunicable diseases (SC-NCDs) globally present a challenge for health systems, particularly in rural settings of low- and lower-middle-income countries (LLMICs). The Package of Essential NCD Interventions-Plus (PEN-Plus) is a strategy currently used in 14 LLMICs to effectively manage people living with SC-NCDs (PLWSC-NCDs), including in Nhamatanda, Sofala Province, Mozambique.

**Objectives:**

We are conducting a cohort study of PLWSC-NCDs, enrolled at Nhamatanda PEN-Plus clinic, to understand their experiences in care and clinic evolution. Here, we describe our methods and the cohort at baseline.

**Methods:**

An 18th month cohort study was initiated shortly after the launch of the PEN-Plus clinic. At baseline, PLWSC-NCD and healthcare providers were enrolled in the study. Qualitative interviews and quantitative measurements including diabetes distress for patients and provider shadowing and task mapping were conducted at baseline and will be carried out semiannually for 18 months.

**Results:**

Twenty-three PLWSC-NCDs and five healthcare providers were enrolled. Most provider training occurred formally pre-service or in service or less formally on job, and providers self-reported varying levels of task proficiencies. Other than personal achievement, we found generally low provider burnout at baseline. We gained insight into patients journey to diagnosis: most patients described a lengthy journey, resulting in unique clinical presentations and associated costs reflected by patients’ borrowing money or selling assets.

**Conclusion:**

There are health systems challenges associated with diagnosis and treatment of SC-NCDs in LLMICs. PEN-Plus is a strategy designed to provide mid-level healthcare workers with the necessary training and tools. This 18-month study will examine the evolution of care at the Nhamatanda clinic.

## Paper Context

- **Main findings:** At initiation of this study, most patients living with severe chronic noncommunicable disease experienced prolonged delays in diagnosis, resulting in distinctive clinical presentations and financial strain, while most providers reported varying levels of task proficiency and generally low levels of burnout.
- **Added knowledge:** We garnered insight into the patients journey to diagnosis prior to the initiation of the PEN-Plus clinic. We gained understanding into the level of training, task proficiency, and well-being of providers that provides us a foundation to assess how the PEN-Plus clinic impacts this cohort’s experiences over the course of this study.
- **Global health impact for policy and action:** Severe chronic noncommunicable diseases pose a growing challenge to health systems globally, particularly in low-and lower-middle-income countries, and PEN-Plus provides a strategy to address this challenge. This study evaluates the evaluation of one PEN-Plus clinic in rural Mozambique.

## Background

In low- and lower-middle-income countries (LLMICs), noncommunicable diseases (NCDs) contribute to a notable and growing portion of the overall disease burden [1]. While often thought to be a disease of the rich, 73% of NCD deaths occurred in low- and middle-income countries [2,3]. Severe chronic NCDs (SC-NCDs) – including type 1 diabetes (T1D), heart failure, and sickle cell disease (SCD) – are a key cause of death and disability in this setting due to limited access to comprehensive care [4]. This growing presence of NCDs in developing countries has made NCDs an increasing priority [5,6].

In Mozambique, NCDs have increasing prominence, with a mortality rate of 779 deaths per 100,000 population and a probability of dying from cardiovascular diseases, cancer, diabetes, or chronic respiratory disease between age 30 and 70 at about 31% [7,8]. In 2006, the Mozambican government made NCDs a priority in their National Health Policy and continued in 2020 with the creation of their National Strategic Plan for Noncommunicable Diseases [5,9]. The Universidade Eduardo Mondlane, with support from the Center for Integration Science at the Brigham and Women’s Hospital, convened a National Commission on Reframing NCDs for Poverty in Mozambique. The result was a wide-ranging report highlighting SC-NCDs as a top priority [10].

The World Health Organization’s (WHO) Package of Essential Noncommunicable Disease (PEN) interventions for primary health care has decentralized services for some NCDs (cardiovascular diseases, type 2 diabetes, chronic respiratory diseases, early cancer detection) to the primary care level, but it does not address SC-NCDs [11]. Diagnosis and care for these conditions are typically limited to tertiary-level facilities and urban areas in LLMICs, resulting in significant gaps in access to care in rural areas. The Package of Essential Noncommunicable Disease Interventions-Plus (PEN-Plus) strategy expands upon PEN to address these gaps by decentralizing care for SC-NCDs in resource-limited health systems. It enables midlevel providers including nurses, medical/clinical officers, and non-specialist doctors to diagnose and manage SC-NCDs at intermediate, first-referral level facilities such as district hospitals, through clinics that provide integrated care for a set of prioritized conditions. PEN-Plus provides services previously limited, in most cases, to the tertiary and higher health system levels. Examples include insulin management for T1D, echocardiography for heart failure, and hydroxyurea for SCD. PEN-Plus was developed in Rwanda in 2006 and later scaled nationally to all district level hospitals in 2015 [12,13], and has been found to be effective, feasible, and affordable [14–16]. In 2022, the World Health Organization’s Regional Office for Africa called for high levels of PEN-Plus initiation by 2030 [17]. Currently, PEN-Plus has been implemented in 14 LLMICs, including Rwanda, Haiti, Malawi, Liberia, Ethiopia, Kenya, Mozambique, Nepal, Sierra Leone, Tanzania, Uganda, Zambia, Zimbabwe, and Chhattisgarh State in India [18].

In the end of 2022, PEN-Plus implementation began in Mozambique at Nhamatanda Rural Hospital [18]. Given the novel design of PEN-Plus in the region and significant investment to expand this strategy, we are conducting an 18-month study on the experience of introducing PEN-Plus, with a focus on the experience of providers and patients. Understanding experience in these settings is crucial as care barriers play a critical role in health and care seeking behaviors [19]. The aim of the overall 18-month study is to understand how chronic outpatient care evolves with the launch of PEN-Plus specifically to i) describe the cost of care and experiences in care of PEN-Plus users and ii) to assess the medium-term provider adoption and fidelity to PEN-Plus protocols. In this manuscript, we present the overall study methodology, describe the patient and provider cohorts, and document the patients’ journeys to diagnosis.

## Methods

### Setting

This study is being conducted at the Nhamatanda Rural Hospital, where PEN-Plus consultations are implemented in the province of Sofala. Nhamatanda Rural Hospital is operated by the Mozambique Ministry of Health and has an approximate catchment area of 318,000 inhabitants. PEN-Plus services started in January of 2022, with the assistance from Universidade Eduardo Mondlane faculty and Instituto Nacional de Saúde researchers, as well as managerial support from Doctors with Africa (CUAMM) and the Mozambique Institute for Health Education and Research.

### PEN-Plus

The establishment of the PEN-Plus at Nhamatanda Rural Hospital included 1) hiring and supervising clinical and nonclinical staff members, 2) ongoing staff training and mentorship, 3) procurement and distribution of equipment, medicines, and supplies, 4) supporting infrastructure improvement for clinic space as appropriate, 5) development and supporting implementation of new clinical and data systems, and 6) strengthening internal referrals with emergency room and other in-patient and outpatient clinics [18].

### 18-month cohort study design

Study procedures include a mix of provider shadowing, qualitative interviews, quantitative surveys and information from patient charts. Participants in the study were recruited into the study in February 2024 and followed up at six-monthly intervals for a period of 18 months. The first round of data collection occurred from February 19 to 27 for the baseline assessment of the study. Observation and data collection continued to occur every 6 months for a period of 18 months until October 2025. Follow-up rounds of data collection with patients focused on 1) experience accessing routine and acute care post-diagnosis, 2) clinical outcomes, and 3) direct and indirect out-of-pocket costs.

People living with SC-NCDs (PLWSC-NCD) (specifically either T1D, severe cardiac conditions (including rheumatic heart disease (RHD) and heart failure) or SCD) and the providers of care were enrolled into the study in accordance with the principles of the Declaration of Helsinki. The exposure of interest in this study was exposure to PEN-Plus. At routine visits, potential participants were approached by clinic-specific data staff to assess their interest in participating. Informed consent was obtained from potential participants at the clinic visit prior to enrollment by trained data collectors. In the case of minors, consent was obtained from parents or guardians, and assent was obtained from those who were underage. Willingness to provide consent did not affect eligibility to receive care, but no research was conducted on individuals who did not provide consent.

### Qualitative interviews

At every data collection point, we conducted qualitative interviews with providers and PLWSC-NCDs. Provider interviews examined clinical responsibilities, self-efficacy, workload, challenges faced, perceptions and use of training, and reflections on changes at the clinic in the prior 6 months. Patient interviews examined experiences in health care and self-management of NCD conditions, as well as how these factors have changed since the initiation of PEN-Plus care, including 1) experiences with healthcare providers, 2) experience with self-management, 3) psychosocial impact of their condition, and 4) economic hardship. Qualitative interviews are conducted by trained researchers using semi-structured tool and took place in the participant’s native language.

### Analysis

This manuscript is the first of a planned series on the study and includes data from the entry into an 18-month convergent design mixed-methods single-arm cohort study focusing on patients and providers engaged in a new PEN-Plus clinic. The overall study will be evaluated using Proctor’s outcomes for implementation research [20]. Future analyses will document the acceptability (consumers, providers), adoption (providers), appropriateness (consumers, providers), fidelity (providers) and sustainability (setting) of the program. We will also examine client and service outcomes (consumers) (Table 1).

**Table 1.** Data collection methods and tools utilized.

		Study time point (months)					
Participant type	Assessment type	0	6	12	18	Tool	Proctor outcomes
Patients	Quantitative	x				Intake Questionnaire	Acceptability, Appropriateness, Costs, Service outcomes, Client outcomes
			x	x	x	Follow-Up Questionnaire	Acceptability, Appropriateness, Costs, Service outcomes, Client outcomes
		x	x	x	x	Problem Areas In Diabetes (PAID) Scale*	Client outcomes
	Qualitative	x	x	x	x	Semi-Structured Interview Tool (Patients)	Acceptability, Appropriateness, Service outcomes, Client outcomes,
Providers	Quantitative	x	x	x	x	Observation Checklist: Insulin-Dependent Diabetes	Fidelity
		x	x	x	x	Observation Checklist: Heart Failure	Fidelity
		x	x	x	x	Task Mapping Questionnaire	Adoption, Appropriateness, Fidelity
		x	x	x	x	Maslach Burnout Inventory	Sustainability
	Qualitative	x	x	x	x	Semi-Structured Interview Tool (Providers)	Acceptability, Adoption, Appropriateness,

*T1D patients only.

#### Current paper

In this paper, we report the findings at baseline including the description of patient and provider cohorts. For providers, we describe the results at baseline from task mapping surveys and from the burnout questionnaire. For PLWSC-NCDs, we describe patient experience including how long it took for them to receive a diagnosis, how many and what types of providers they saw, how many hospitalizations they had, and how they paid for their care. Additionally, for PLWT1D, we include baseline diabetes distress.

### Research team

Three data collectors with medical background were selected and trained to collect data, including a field supervisor with experience in qualitative research. The training of the data collectors took place over 2 days in February 2024, at the Nhamatanda Rural Hospital, and included topics such as: ethics in research on human beings, good clinical practice, informed consent assent for minors under 18 years principles of qualitative and quantitative research. The study protocol was also discussed/reviewed emphasizing the study objectives and methodologies proposed, involving both theoretical and practical components of data collection tools. Data collection instruments and procedures were piloted at a nearby satellite health center (Lamego Health Center), which was intentionally selected as it does not yet offer PEN-Plus services.

### Study participants

The study includes patients receiving healthcare services at Nhamatanda Hospital and providers responsible for providing PEN-Plus services. Healthcare providers involved in the provision of care for people enrolled at the PEN-Plus clinic were invited to participate. In parallel, patients presenting to the PEN-Plus clinic were also invited to participate. As T1D has been identified as a tracer condition for PEN-Plus clinics, patients presenting with T1D were oversampled for this element of the study. In Mozambique, general practitioners are defined as professionals who have a degree in General Medicine, and medical technicians are defined as clinicians with secondary medical technician training, in accordance with technical and professional education regulations.

### Data collection overview

Data collection methods and tools utilized varied by participant type (Table 1). All tools were translated to Portuguese prior to use and adapted after piloting. Quantitative tools were administered using REDCap (Research Electronic Data Capture), an electronic data capture tool hosted at Mass General Brigham [21,22]. This paper focuses on describing the enrolled cohort at baseline.

### Provider data collection for

We used a mixed-methods approach consisting of quantitative assessments and semi-structured qualitative interviews. Over the course of one-week, trained data collectors shadowed providers at the PEN-Plus clinic, observing consultations of patients with T1D and/or heart failure, and documenting providers’ performance of specific tasks using observation checklists. Providers were shadowed from 7:30 until 15:30 for 5 days. Quantitative task mapping surveys assessed providers’ key activities at the PEN-Plus clinic. Providers were asked to assess four domains of each activity: 1) Frequency – how often the provider performs the task, 2) Criticality – the provider’s view on the cruciality of timely and effective completion of this task to patient outcomes, 3) Training – when and where the provider was trained to perform the task (if at all), and 4) Performance – the provider’s perceived competence in their performance of the task [23]. Providers could indicate multiple periods when they received training on a particular task, which could be pre-service (trained to complete task as part of pre-service education), in-service (trained to complete task following graduation), and/or on the job (received informal training from co-workers or supervisor). The providers rated their level of competence on a task from not competent (cannot perform the task safely without assistance and may cause harm if performing the task without supervision), competent (can perform the task safely and effectively and may ask for assistance as necessary), to proficient (proficient and may instruct others in performing the task).

Providers were asked to complete the Maslach Burnout Inventory [24], a widely used measure of occupational burnout focusing on emotional exhaustion, depersonalization, and personal accomplishment in the workplace.

### Patient data collection

We used quantitative surveys conducted at enrollment to detail PLWSC-NCD journey to diagnosis, including catastrophic costs, hospitalizations and symptoms. These tools were created by our team based on prior research conducted by our team [25]. In each encounter T1D patients are asked to complete the Problem Areas In Diabetes (PAID) Scale, a validated 20-item instrument used to measure diabetes-related distress [26].

### Data analysis

We used descriptive statistics to analyze quantitative variables. For providers, results are shown both individually and pooled. For PLWSC-NCDs, we report findings pooled by condition, and overall. We used a conversion of 63.3 Metical to one USD based on 31 March 2024 exchange rates [27].

Qualitative data will be transcribed and translated into English. Future analysis will be done using deductive approach using a-priori themes based on the study proposal and Proctor outcomes. Emergent themes will be added to the term list, and interviews will be recoded to ensure all codes are utilized.

## Results

### Baseline status of providers

We enrolled two general practitioners, two medical technicians/clinical officers and one nurse. Table 2 shows their training and self-perceived competence for specified tasks related to cardiac conditions (including RHD and heart failure), T1D, and sickle cell disease. All providers received training around cardiac conditions, T1D and SCD at some point in time; two providers did not receive any training in anticoagulation management (one physician and one non physician, Appendix 1, Appendix 2), but they do not provide this service at the clinic. Training for highlighted conditions occurred pre-service, in-service and on the job, and providers reported having received some training for all competencies except for anti-coagulation management. Providers felt varying levels of proficiencies across tasks but overall felt most proficient conducting tasks associated with T1D, and least proficient around cardiac conditions. Our results suggest that no providers had high-level burnout in either the burnout or depersonalization categories, but two scored high-level burnout in the personal achievement categories (Table 3).

**Table 2.** Provider training and performance of tasks at baseline.

Task	Training (could report multiple training events)				Performance (N = 5)		
	Pre-service (n)	In-service (n)	On the job (n)	Not Trained (n)	Not Competent (n)	Competent (n)	Proficient (n)
**Cardiac**							
Acquisition of echocardiographic images	2	4	4	0	2	1	2
Interpretation of echocardiograms	2	3	3	0	2	2	1
Fluid status management	4	4	2	0	0	2	3
Beta-blocker and ACE inhibitor management	4	5	2	0	0	2	3
Anticoagulation (warfarin) management	2	3	0	2	2	2	1
**Type 1 Diabetes**							
Insulin management	4	5	3	0	0	2	3
Foot exam	4	4	3	0	0	1	4
Interpretation of home glucose testing	4	3	2	0	0	0	5
Complications screening	4	4	4	0	0	0	5
Conducting self-management education	4	4	2	0	0	2	3
**Sickle Cell Disease**							
Penicillin prophylaxis management	4	4	3	0	0	2	3
Initiation and monitoring of hydroxyurea	3	3	3	0	0	3	2
Pain management, opioids	4	4	3	0	0	2	3
Complications screening	3	3	3	0	0	2	3

Training: When and where educated/trained to perform the task. Pre-service education: Trained to complete task as part of pre-service education. In-service education: Educated/trained to complete task following graduation. On the job: Received informal education/training from co-workers or supervisor. Not trained: Did not receive any pre-service, in-service, or on-the-job training for the task.

Performance: Level of competence in performing the task. Proficient: Proficient and may instruct others in the performance of the task. Competent: Perform the task safely and effectively; May ask for assistance as necessary. Not competent: Cannot perform the task safely without assistance and may cause harm if performing the task without supervision.

**Table 3.** Provider Maslach Burnout Inventory (MBI) at baseline.

Maslach Burnout Inventory Component	Providers (*N* = 5)
**Burnout**	
High-level burnout (30+)	0
Moderate burnout (18–29)	1
Low level-burnout (17 or less)	4
**Depersonalization**	
High-level burnout (12+)	0
Moderate burnout (6–11)	2
Low level-burnout (5 or less)	3
**Personal Achievement**	
Low-level burnout (40+)	2
Moderate burnout (34–39)	1
High level-burnout (33 or less)	2

Burnout (or depressive anxiety syndrome): Testifies to fatigue at the very idea of work, chronic fatigue, trouble sleeping, physical problems. For the MBI, as well as for most authors, ‘exhaustion would be the key component of the syndrome.’ Unlike depression, the problems disappear outside work. Depersonalisation” (or loss of empathy): Rather a ‘dehumanisation’ in interpersonal relations. The notion of detachment is excessive, leading to cynicism with negative attitudes with regard to colleagues, feeling of guilt, avoidance of social contacts and withdrawing into oneself. The professional blocks the empathy they can show to their colleagues. The reduction of personal achievement: The individual assesses themselves negatively, feels they are unable to move the situation forward. This component represents the demotivating effects of a difficult, repetitive situation leading to failure despite efforts. The person begins to doubt their genuine abilities to accomplish things. This aspect is a consequence of the first two [24].

### Baseline data for people living with SC-NCDs

At the PEN-Plus clinic in February of 2024, there were 61 patients eligible to join this study, including 20 people living with T1D,19 people living with sickle cell disease, 14 people living with heart failure and eight people living with RHD. Of these, we enrolled 23 patients (14 women and nine men-12 living with T1D, four living with SCD, five living with heart failure, and two living with RHD). Patient participants ranged in age from 11 to 79 years, with an overall mean of 29. Most patients (60.9%) had either secondary education or an apprenticeship (Table 4).

**Table 4.** Patient characteristics by condition.

Characteristic		Condition				
		*T1D* *n = 12*	*SCD* *n = 4*	*RHD* *n = 2*	*HF* *n = 5*	*Total* *n = 23*
Person interviewed (n (%))	Self	11 (91.7)	0 (0.0)	2 (100.0)	3 (60.0)	16 (69.6)
	Self + Guardian	1 (8.3)	4 (100.0)	0 (0.0)	1 (20.0)	6 (26.1)
	Guardian	0 (0.0)	0 (0.0)	0 (0.0)	1 (20.0)	1 (4.3)
Sex (n (%))	Female	5 (41.7)	4 (100.0)	1 (50.0)	4 (80.0)	14 (60.9)
	Male	7 (58.3)	0 (0.0)	1 (50.0)	1 (20.0)	9 (39.1)
Age (years)	Mean	26	14	23	50	29
	Median	27	14	23	59	26
	Range	16–39	11–16	14–32	13–79	11–79
Highest level education (n (%))	None	0 (0.0)	0 (0.0)	0 (0.0)	1 (20.0)	1 (4.3)
	Primary	3 (25.0)	1 (25.0)	1 (50.0)	3 (60.0)	8 (34.8)
	Secondary	7 (58.3)	3 (75.0)	1 (50.0)	1 (20.0)	12 (52.2)
	Apprenticeship	2 (16.7)	0 (0.0)	0 (0.0)	0 (0.0)	2 (8.7)
Self-reported literacy (n (%))	Yes	10 (83.3)	3 (75.0)	2 (100.0)	2 (40.0)	17 (73.9)
	No	2 (16.7)	1 (25.0)	0 (0.0)	3 (60.0)	6 (26.1)
Marital status (n (%))	Single	8 (66.7)	4 (100.0)	1 (50.0)	2 (40.0)	15 (65.2)
	Monogamously married	4 (33.3)	0 (0.0)	1 (50.0)	2 (40.0)	7 (30.4)
	Widowed	0 (0.0)	0 (0.0)	0 (0.0)	1 (20.0)	1 (4.3)

Most people living with SC-NCDs in this study had a lengthy journey to diagnosis. The overall range from first noticing symptoms to diagnosis was between 3 days and over 14 years. A patient’s journey to receiving a diagnosis differed depending upon their condition. For these patients, the average amount of time from when they first observed symptoms to when they sought care was 337 days, with a median of 60 days (Table 5). Patients with heart failure had the longest median time to seek care (180 days), while those with SCD had the shortest (3 days). Patients with T1D self-reported the widest range, with some seeking care as quickly as 1 day while another patient reported it took up to 5110 days.

**Table 5.** Characteristics of patient journey to diagnosis by condition.

Characteristic		Condition				
		*T1D* *n = 12*	*SCD* *n = 4*	*RHD* *n = 2*	*HF* *n = 5*	*Total* *n = 23*
Length of time patient first observed symptoms before seeking care (days)	Mean	517	93	57	213	337
	Median	45	3	57	180	60
	Range	1–5110	2–365	30–84	35–365	1–5110
Length of time between seeking care and receiving diagnosis (days)	Mean	17	640	17	32	129
	Median	6	730	17	7	7
	Range	1–60	3–1095	4–30	1–120	1–1095
Length of time between first observing symptoms and diagnosis (days)	Mean	534	733	74	245	466
	Median	77	914	74	181	120
	Range	3–5170	6–1098	60–88	65–485	3–5170
Health providers visited prior to diagnosis*	Herbalist/Traditional	4	1	1	1	7
	Religious leader	1	3	0	2	6
	Nurse	11	4	1	3	19
	Doctor	4	1	1	2	8
	Clinical officer	0	0	0	1	1
	Physician assistant	3	1	0	1	5
	Pharmacist	3	1	0	1	5
	Other	1	0	0	0	1
	Total	27	11	3	11	52
Health facilities visited prior to diagnosis*	Central hospital	3	1	2	0	6
	District hospital	8	4	1	2	15
	Health center	5	2	1	4	12
	Private pharmacy, drug or grocery store	1	0	0	0	1
	Other	1	0	0	1	2
	Total	18	7	4	7	36
Comorbidity (n (%))	Yes	4 (33.3)	0 (0.0)	0 (0.0)	3 (60.0)	7 (30.4)
	No	8 (66.7)	4 (100.0)	2 (100.0)	2 (40.0)	16 (69.6)
Comorbidities*	Chronic heart disease	0	0	0	2	2
	Hypertension	3	0	0	1	4
	Liver failure/cirrhosis	0	0	0	1	1
	Other	2	0	0	1	3
	Total	5	0	0	5	10
Prior diagnosis (n (%))	Yes	2 (16.7)	2 (50.0)	0 (0.0)	2 (40.0)	6 (26.1)
	No	10 (83.3)	2 (50.0)	2 (100.0)	3 (60.0)	17 (73.9)
Number of lifetime hospitalizations	Mean	2	3	1	2	2
	Median	1	3	1	1	1
	Range	0–6	1–7	1	1–4	0–7
Covering Costs for NCD Illness						
Borrowed money (n (%))	Yes	8 (66.7)	1 (25.0)	1 (50.0)	3 (60.0)	13 (56.5)
	No	4 (33.3)	3 (75.0)	1 (50.0)	2 (40.0)	10 (43.5)
Amount borrowed (Metical) [USD $]**	Mean	13925 [220.1]	5000 [79.0]	500 [7.9]	3000 [47.4]	9685 [153.1]
	Median	2000 [31.6]	5000 [79.0]	500 [7.9]	3000 [47.4]	2000 [31.6]
	Range	500–80000 [7.9–1264.6]	5000 [79.0]	500 [7.9]	1000–5000 [15.8–79.0]	500–80000 [7.9–1264.6]
Borrowed from ^Ɨ^	Family	3	1	0	3	7
	Neighbors/Friends	6	1	1	1	9
	Cooperative	1	0	0	0	1
	Other	1	0	0	0	1
	Total	11	2	1	4	18
Sold assets (n (%))	Yes	7 (58.3)	3 (75.0)	1 (50.0)	3 (60.0)	14 (60.9)
	No	5 (41.7)	1 (25.0)	1 (50.0)	2 (40.0)	9 (39.1)
Assets sold: livestock ^Ɨ^	Poultry	1	0	0	2	3
	Goat	1	0	0	1	2
	Total	2	0	0	3	5
Assets sold: other ^Ɨ^	Household Item	2	0	0	0	2
	Farm produce	2	3	1	2	8
	Other	1	1	0	1	3
	Total	5	4	1	3	13
Borrowed money or sold assets (n (%))	Yes	11 (91.7)	3 (75.0)	2 (100.0)	5 (100.0)	21 (91.3)
	No	1 (8.3)	1 (25.0)	0 (0.0)	0 (0.0)	2 (8.7)

Abbreviations: T1D, Type 1 Diabetes; SCD, Sickle Cell Disease; RHD, Rheumatic Heart Disease; HF, Heart Failure.

*Patients could report multiple provider and facility types, including several within one type, as well as more than one comorbidity.

**Of those who borrowed money (*n* = 13). Metical to USD (63.3 Metical = 1 USD), exchange rate based on 03/31/2024 U.S Treasury foreign currency rates [27].

^Ɨ^Patients were able to report multiple types of assets sold and multiple sources from which they borrowed money.

After seeking care, the average time it took for patients to receive a diagnosis was 129 days, with a median of 7 days. Patients with SCD experienced the longest delays, ranging from 3 to 1095 days, with a median time of 730 days to receive a diagnosis. Prior to receiving a diagnosis, the most common type of health provider patients visited were nurses (19), with patients living with T1D accounting for most of these visits (11). These patients also accounted for most visits to district hospitals (8 out of 15), the most frequently visited type of health facility. Most PLWSC-NCDs reported being hospitalized at least once for their condition (87%, Tables 5 and 6).

**Table 6.** (continued).

Participant	Journey to diagnosis					Lifetime hospitalizations for NCD
	Approx. duration between observing symptoms and seeking care (days)	Approx. duration between seeking care and diagnosis (days)	Approx. duration between observing symptoms and diagnosis (days)	# facilities visited prior to diagnosis	# providers seen prior to diagnosis	
Heart failure						
HF1	35	30	65	1	3	1
HF2	365	122	487	2	1	4
HF3	365	3	368	2	3	1
HF4	122	7	129	1	3	1
HF5	183	1	184	1	1	2
Rheumatic heart disease						
RHD1	30	30	60	3	2	1
RHD2	84	4	89	1	1	1
Sickle cell disease						
SC1	2	1095	1097	1	1	3
SC2	3	3	6	2	2	1
SC3	365	365	730	2	2	2
SC4	3	1095	1098	2	6	7
Type 1 diabetes						
T1D1	28	28	56	1	1	2
T1D2	5110	61	5171	1	2	2
T1D3	21	1	22	2	2	0
T1D4	30	4	34	1	1	1
T1D5	30	7	37	1	3	1
T1D6	92	30	122	3	2	1
T1D7	92	7	99	1	4	0
T1D8	365	1	366	1	3	0
T1D9	365	4	369	1	2	6
T1D10	1	2	3	3	1	2
T1D11	14	1	15	2	3	1
T1D12	61	61	122	1	2	3

**Table 6.** Patient pathways to diagnosis.

Participant	Date of diagnosis	Age (years)	Symptom																					
			Chest pain	Palpitations	Blurred vision	Dizziness	Syncope	Headache	Change in appetite	Numbness	Night sweats	Unexpected weight changes	Swelling legs	Shortness of breath	Coughing	Abdominal pain	Nausea/vomiting	Abdominal swelling	Difficulty walking	Frequent urination	Thirst	Fatigue	Weakness	Rash or other skin problems
Heart failure																								
HF1	2/19/2024	65											x			x						x	x	
HF2	2/19/2024	35	x					x					x	x										
HF3	10/3/2023	59	x	x	X				x			x	x	x	x	x	x						x	
HF4	10/19/2023	79	x	x			x															x	x	
HF5	3/18/2011	13		x																		x	x	
Rheumatic heart disease																								
RHD1	2/23/2023	14		x					x				x	x					x			x	x	
RHD2	12/22/2020	32	x											x								x		
Sickle cell disease																								
SC1	11/21/2017	14		x				x	x							x			x			x	x	
SC2	9/29/2023	11	x	x				x	x					x								x		
SC3	9/6/2023	16		x				x	x		x		x		x		x		x					
SC4	2/21/2024	14	x						x			x		x								x		
Type 1 diabetes																								
T1D1	15/6/2013	26		x	X	x		x	x	x	x	x				x		X	x	X	x	x	x	x
T1D2	03/01/2010	39			X							x		x						X	x		x	
T1D3	8/25/2023	16		x	X	x			x	x		x								X	x			
T1D4	8/23/2023	18		x	X	x	x	x	x	x	x	x		x					x	X	x	x	x	x
T1D5	10/30/2023	24								x		x			x					X	x	x	x	
T1D6	8/17/2016	35			X	x		x	x	x	x	x	x	x						X	x	x	x	
T1D7	4/6/2023	31						x	x	x		x				x					x		x	
T1D8	8/15/2018	31			X				x		x	x								X	x		x	
T1D9	10/24/2019	27			X	x			x			x								X	x		x	
T1D10	1/1/2019	27	x					x						x					x			x		
T1D11	8/3/2023	16						x	x	x		x			x					X		x		
T1D12	1/8/2017	20																		X	x	x		

(*Continued*)

Most patients (69.6%) did not self-report living with a comorbid condition. Before receiving their current NCD diagnosis, most patients (73.9%) did not have a diagnosis for a different condition. Only six of the 23 patients (26.1%) indicated that they had a prior diagnosis. Among the 30.4% of patients living with a comorbid condition at the time of the study, hypertension was the most commonly reported comorbidity (17.4%), with 75% of patients with hypertension also living with T1D. Patients with heart failure reported a variety of comorbid conditions, including chronic heart disease (2), hypertension (1), liver failure/cirrhosis (1), and/or other illnesses (1). There was no obvious pattern between age and presence of a comorbidity, possibly due to low sample sizes.

To cover the costs of an NCD illness, 91.3% of patients borrowed money or sold their assets (Table 5). Just over half (56.5%) of patients reported borrowing money to cope with the cost of their NCD illness, with a mean of 9685 [$153.1] and a median of 2000 Metical, 31.6 USD. Neighbors and friends were the most common sources of borrowed funds. Other sources included family, cooperatives, and a timber company.

Selling assets was another means for patients to cover the cost of their NCD illness, with a higher proportion, 60.9% (14) of all patients reporting that they have sold assets. Of those who sold assets, poultry (3) was the most common livestock asset sold, while farm produce (8) was the most common type of non-livestock asset sold. Two participants reported selling goats. Patients living with sickle cell disease were more likely to sell assets (3 out of 4, 75%), with farm produce (3) being the most frequently type of assets sold (Table 5).

## Discussion

We describe the methodology and cohort for an 18-month mixed-methods longitudinal study of 23 PLWSC-NCDs enrolled at a rural hospital and five healthcare providers responsible for their care in that rural facility. Provider training occurred formally in pre-service, in-service or less formally on the job, and providers self-reported varying levels of proficiencies across tasks. Most patients described a lengthy journey to diagnosis, resulting in unique clinical presentations that reflect delayed diagnosis with complications, and associated costs. Linked to this, a large proportion of patients self-report needing to either borrow money or sell assets to cover the cost of care.

All providers received training in tasks for cardiac conditions (including RHD and heart failure), T1D, and SCD at some point in time; the two providers who did not receive any training in anticoagulation management were not involved in this task. The self-perceived level of competence providers had in performing procedures was lowest on tasks related to cardiac conditions. This is consistent with a study showing that midlevel health workers were poorly equipped to treat RHD in pregnant women [28]. In contrast, most providers perceived themselves as proficient in tasks associated with T1D. We found no discernable trends based on the cadre of providers and their self-perceived competency in performing these tasks. However, as competencies are self-reported, providers are likely to report differing levels of competencies depending on the flow of patients they are seeing. A provider seeing relatively complex patients may report lower competencies based on the complexity of the case than a provider seeing relatively simpler patients regardless of their overall competency. Reflecting upon these differences when training occurs, its frequency, and perceived level of competence in performing specific tasks opens the discussion on ways to improve areas of low provider competency and maintain high competency levels in performing different tasks. While task-shifting can be complex for some conditions under investigation here, our results cautiously suggest that care can be safely task-shifted. Overall, our study found low levels of provider burnout at baseline, except for personal achievement, where two providers exhibited high levels of burnout. Future analyses will specifically track changes in competencies and burnout throughout the evolution of the PEN-Plus clinic, as we track providers for 18 months.

Patients reported a lengthy time between initiation of symptoms and diagnosis of disease, with many patients waiting years for diagnosis. This delayed diagnosis [29,30] likely stems from a variety of factors including the breadth of non-specific symptoms that PLWSC-NCDs present with and health system challenges and low clinical suspicion linked to rarity of these more serious NCDs. Participants in this study reported a variety of symptoms across diseases, some of which may not be diagnostic of the particular disease that they actually had, some of this is likely due to comorbidities, however PLWSC-NCDs in rural settings of Mozambique in general have low literacy, low exposure to health services, and often will attribute diseases to outside causes. This can affect recall bias through the misattribution of health symptoms to events or interactions with community members. PLWSC-NCDs in this study presented with very non-specific systems. This has profound implications for case-finding efforts throughout similar settings.

The lack of medicines, diagnostic tools, and supplies severely hinders appropriate NCD diagnosis and care. These findings are consistent with other studies conducted in Mozambique and elsewhere in [19,25,31,32] the region that examine care pathways for patients with severe disease. Barriers to diagnosis and management stem from an array of factors [33]. Delayed recognition on the parts of patients and, limited sensitization and misdiagnosis by providers, and widespread gaps in necessary diagnostic kits and consumables all play a role in delayed diagnosis. Healthcare providers and managers interviewed in other studies emphasized the need for greater availability of these resources alongside expanded NCD-specific training to build their capacity to manage NCDs [34,35].

Studies have identified a lack of preparation among healthcare providers in addressing NCDs at peripheral health facilities, with some showing the potential of non-specialists to assess, diagnose, and manage a subset of conditions (hypertension, diabetes, and cardiovascular risk) to support the decentralization of NCD care [36]. In our cohort, nurses were the most visited health providers before diagnosis, and district hospitals were the most frequently utilized facilities. While patients tried visiting lower-level facilities early in their journey, they left unsatisfied and need to seek out care at higher levels, and needed to come to a district level hospital to get diagnosis. This finding is as expected given the health systems challenges currently in Mozambique, where there is an absence of primary care for complex diseases. At lower-level facilities, low- and mid-level providers, including nurses and medical officers, are the only available qualified health care providers. Many patients find it necessary to travel to district or central hospitals to obtain proper NCD diagnoses. Equipping healthcare providers with targeted NCD training could help reduce delays in care by increasing their competency in performing NCD-related tasks and enhancing their ability to identify, diagnose, treat and, critically, to refer patients needing higher levels of care. PEN-Plus addresses these overall health system challenges by strengthening internal referrals with emergency rooms, in-patient services and other outpatient clinics, to familiarize providers in these settings with the diagnostic signs and initial management of these NCDs. Additionally, through forging referral and counter referral links with satellite primary healthcare centers, the PEN-Plus clinic helps ensure patient retention in care and increase the visibility of NCDs.

The heavy toll of this delayed diagnosis and management on our participants is evidenced by the high rate of hospitalizations, borrowed money and sold assets. Efforts to maintain patients in care are hindered by the supply-side challenges highlighted above, including frequent stock outs of medications, as well as considerable physical and financial access barriers faced by patients. These challenges are exacerbated by limited patient health literacy. Highlighting the complexity of health care communication, experience has shown that loss to follow-up can occur both when patients do and do not perceive improvements in their health status.

Public sector healthcare facilities, typically the first point of care for patients seeking subsidized services, often struggle to meet patients’ needs due to high caseloads, limited infrastructure, and a lack of diagnostic technology and essential medicines for NCDs. As a result, this forces many patients to seek care in the private sector, where they must pay out-of-pocket – an option that can be prohibitively expensive. For instance, one study found that the lowest-paid worker in Mozambique would need to spend 14 to 17.8 days’ wages to afford a month’s supply of cardiovascular disease medicine [37]. Similarly, in our study, the prohibitive cost of care was evident, as over 91% of PLWSC-NCDs had to borrow money and/or sell assets to access necessary healthcare, and the high rate of hospitalization added to these costs.

This study has several limitations; The small sample size precludes understanding of the differences between patient journeys by disease, provider training and any formal statistical analysis. Our study relies completely on self-reported data, so is particularly subject to recall bias and social desirability bias. We acknowledge that some patient self-reported data are unrealistic, for example taking 15 years to obtain a T1D diagnosis. Despite these limitations, the study addresses key aspects of chronic care in resource-constrained settings, that have not been systematically evaluated and reported but are key to the choice of design of models for care for chronic diseases in LLMICs.

By decentralizing care for SC-NCDs in resource-limited health systems, the PEN-Plus strategy addresses some of these healthcare challenges. It enables midlevel providers like nurses, medical officers and non-specialist doctors to diagnose and manage SC-NCDs through integrated clinics at intermediate first-referral level facilities, such as Nhamatanda Rural Hospital [12–16,18]. Before initiating this PEN-Plus initiative, Nhamatanda District Hospital had a limited capacity to diagnose and treat patients with SC-NCDs, having only a handful with T1D and RHD, and no patients with SCD [38]. This study will provide longitudinal data to enable us to track providers’ skill levels and obtain self-reported confidence while tracking to ensure they are not experiencing burnout. At the same time, we will monitor patients to ensure they receive good-quality patient-centered care and that their needs and values are being considered.

## Conclusions

This work presents the study design and cohort details designed to document the launch of an integrated PEN-Plus clinic. Patients enrolling in the study documented significant challenges in obtaining a diagnosis for SC-NCDs in rural Mozambique, describing very long journeys to diagnosis associated with large out-of-pocket costs and multiple hospitalizations. There are substantial health systems challenges around the diagnosis and treatment of SC-NCDs at secondary level facilities in Mozambique and other LLMICs, and providers in this study self-reported variable levels of training and ability to perform tasks. The PEN-Plus strategy is one strategy designed to decentralize care to secondary-level facilities and provide midlevel healthcare workers the training and tools needed. This 18-month study will examine the evolution of care at this clinic.

## Supplementary Material

STROBE_checklist_Nhamatanda.docx

Appendix 1 and 2.docx

## Data Availability

Data and materials supporting the results will be made available upon reasonable request.
