# Supplementary material for: Patient and provider experiences with PEN-Plus in rural Mozambique: cohort characteristics at baseline
Source: Glob Health Action. 2026 Mar 2;19(1):2623347. doi: 10.1080/16549716.2026.2623347 (PMC12954810; doi:10.1080/16549716.2026.2623347)
Supplement: Appendix 1 and 2.docx [file ZGHA_A_2623347_SM0141.docx]

Appendix 1: Provider Training and Performance of Tasks at Baseline-Non-Physicians.

| **Task** | **Training (could report multiple training events)** | | | | | **Performance (N=3) *** | | |
| --- | --- | --- | --- | --- | --- | --- | --- | --- |
|  | **Pre-service (n)** | **In-service (n)** | | **On the job (n)** | **Not Trained (n)** | **Not Competent (n)** | **Competent (n)** | **Proficient (n)** |
| **Cardiac** | | | | | | | | |
| Acquisition of echocardiographic images | 1 | 2 | 2 | | 0 | 1 | 1 | 1 |
| Interpretation of echocardiograms | 1 | 1 | 2 | | 0 | 1 | 2 | 0 |
| Fluid status management | 2 | 2 | 2 | | 0 | 0 | 2 | 1 |
| Beta-blocker and ACE inhibitor management | 2 | 3 | 1 | | 0 | 0 | 2 | 1 |
| Anticoagulation (warfarin) management | 1 | 2 | 0 | | 1 | 1 | 1 | 1 |
| **Type 1 Diabetes** | | | | | | | | |
| Insulin management | 2 | 3 | 1 | | 0 | 0 | 2 | 1 |
| Foot exam | 2 | 2 | 2 | | 0 | 0 | 1 | 2 |
| Interpretation of home glucose testing | 2 | 2 | 2 | | 0 | 0 | 0 | 3 |
| Complications screening | 2 | 2 | 2 | | 0 | 0 | 0 | 3 |
| Conducting self-management education | 2 | 2 | 2 | | 0 | 0 | 2 | 1 |
| **Sickle Cell Disease** | | | | | | | | |
| Penicillin prophylaxis management | 2 | 2 | 2 | | 0 | 0 | 2 | 1 |
| Initiation and monitoring of hydroxyurea | 1 | 1 | 2 | | 0 | 0 | 3 | 0 |
| Pain management, opioids | 2 | 2 | 2 | | 0 | 0 | 2 | 1 |
| Complications screening | 1 | 1 | 2 | | 0 | 0 | 2 | 1 |
| Training: When and where educated/trained to perform the task   - Pre-service education: Trained to complete task as part of pre-service education - In-service education: Educated/trained to complete task following graduation - On the job: Received informal education/training from co-workers or supervisor - Not trained: Did not receive any pre-service, in-service, or on-the-job training for the task   Performance: Level of competence in performing the task   - Proficient: Proficient and may instruct others in the performance of the task - Competent: Perform the task safely and effectively; May ask for assistance as necessary - Not competent: Cannot perform the task safely without assistance and may cause harm if performing the task without supervision   * 2 clinical officers, and 1 nurse | | | | | | | | |

Appendix 2: Provider Training and Performance of Tasks at Baseline-Physicians

| **Task** | **Training (could report multiple training events)** | | | | **Performance (N=2) *** | | |
| --- | --- | --- | --- | --- | --- | --- | --- |
|  | **Pre-service (n)** | **In-service (n)** | **On the job (n)** | **Not Trained (n)** | **Not Competent (n)** | **Competent (n)** | **Proficient (n)** |
| **Cardiac** | | | | | | | |
| Acquisition of echocardiographic images | 1 | 2 | 2 | 0 | 1 | 0 | 1 |
| Interpretation of echocardiograms | 1 | 2 | 1 | 0 | 1 | 0 | 1 |
| Fluid status management | 2 | 2 | 0 | 0 | 0 | 0 | 2 |
| Beta-blocker and ACE inhibitor management | 2 | 2 | 1 | 0 | 0 | 0 | 2 |
| Anticoagulation (warfarin) management | 1 | 1 | 0 | 1 | 1 | 1 | 0 |
| **Type 1 Diabetes** | | | | | | | |
| Insulin management | 2 | 2 | 2 | 0 | 0 | 0 | 2 |
| Foot exam | 2 | 2 | 1 | 0 | 0 | 0 | 2 |
| Interpretation of home glucose testing | 2 | 1 | 0 | 0 | 0 | 0 | 2 |
| Complications screening | 2 | 2 | 2 | 0 | 0 | 0 | 2 |
| Conducting self-management education | 2 | 2 | 0 | 0 | 0 | 0 | 2 |
| **Sickle Cell Disease** | | | | | | | |
| Penicillin prophylaxis management | 2 | 2 | 1 | 0 | 0 | 0 | 2 |
| Initiation and monitoring of hydroxyurea | 2 | 2 | 1 | 0 | 0 | 0 | 2 |
| Pain management, opioids | 2 | 2 | 1 | 0 | 0 | 0 | 2 |
| Complications screening | 2 | 2 | 1 | 0 | 0 | 0 | 2 |
| Training: When and where educated/trained to perform the task   - Pre-service education: Trained to complete task as part of pre-service education - In-service education: Educated/trained to complete task following graduation - On the job: Received informal education/training from co-workers or supervisor - Not trained: Did not receive any pre-service, in-service, or on-the-job training for the task   Performance: Level of competence in performing the task   - Proficient: Proficient and may instruct others in the performance of the task - Competent: Perform the task safely and effectively; May ask for assistance as necessary - Not competent: Cannot perform the task safely without assistance and may cause harm if performing the task without supervision   * 2 general practitioners | | | | | | | |
